# Rapid model building of α-helices in electron-density maps

**DOI:** 10.1107/S0907444910000314

**Published:** 2010-02-12

**Authors:** Thomas C. Terwilliger

**Affiliations:** a Los Alamos National Laboratory, Los Alamos, NM 87545, USA

**Keywords:** structure solution, model building, Protein Data Bank, α-helices, *PHENIX*, experimental electron-density maps

## Abstract

A method for the identification of α-helices in electron-density maps at low resolution followed by interpretation at moderate to high resolution is presented. Rapid identification is achieved at low resolution, where α-helices appear as tubes of density. The positioning and direction of the α-helices is obtained at moderate to high resolution, where the positions of side chains can be seen. The method was tested on a set of 42 experimental electron-density maps at resolutions ranging from 1.5 to 3.8 Å. An average of 63% of the α-helical residues in these proteins were built and an average of 76% of the residues built matched helical residues in the refined models of the proteins. The overall average r.m.s.d. between main-chain atoms in the modeled α-helices and the nearest atom with the same name in the refined models of the proteins was 1.3 Å.

## Abstract 2

A method for rapid model building of α-helices at moderate resolution is presented.

## 1. Introduction   

Building an atomic model is a key step in the interpretation of electron-density maps of macromolecules. Atomic models can be simple and readily visualized representations of the structures of macromolecules and are commonly used as the primary means of conveying structural information about a macromolecule.

Many methods have been developed for manual, semi-automatic and automatic interpretation of electron-density maps from macromolecules. Interactive methods include manual building of models into maps [*e.g. O* (Jones *et al.*, 1991 ▶), *MAIN* (Turk, 1992 ▶), *XtalView* (McRee, 1999 ▶) and *Coot* (Emsley & Cowtan, 2004 ▶)] as well as on-demand local interpretation of maps in which the user specifies some information about the chain location or geometry and a model is automatically generated (Oldfield, 1994 ▶; Jones & Kjeldgaard, 1997 ▶; McRee, 1999 ▶). There are also a number of highly automated methods for the interpretation of maps of proteins. These include procedures for the identification of C^α^-atom positions followed by the generation of complete polypeptide chains (Oldfield, 2002 ▶, 2003 ▶; Ioerger & Sacchettini, 2003 ▶; Cowtan, 2006 ▶), methods focusing on the identification of helical and extended structures followed by tracing loops and other structure (Levitt, 2001 ▶; Terwilliger, 2003 ▶), methods based on the identification of atomic positions and their interpretation in terms of a polypeptide chain (Perrakis *et al.*, 1999 ▶), methods that use extensive conformational sampling (DePristo *et al.*, 2005 ▶), probabilistic methods based on the recognition of density patterns in electron-density maps (DiMaio *et al.*, 2007 ▶) and methods analyzing lower resolution density features in maps (Baker *et al.*, 2007 ▶).

While these are powerful tools for the automated interpretation of electron-density maps representing structures of proteins, they typically take considerably longer to carry out than other initial steps in structure determination (heavy-atom location, phasing and density modification). Additionally, they all become progressively less effective as the resolution of the map decreases, although some progress has recently been made in this regard (DiMaio *et al.*, 2007 ▶).

One approach for speeding up map interpretation and for broadening the resolution range over which accurate model building can be carried out is to identify and interpret features in the map that are as large as possible. In this way a sub­stantial portion of a model can be generated all at once. Furthermore, provided that the features that are identified in this way are relatively uniform over many structures, these features can potentially be modelled accurately. The experience of many crystallographers has demonstrated that α-­helices can readily be identified at low (5–8 Å) resolution (DeLaBarre & Brunger, 2006 ▶). At higher resolution, the *O* software has shown that the direction (and placement) of α-­helices in a map can be accurately identified by averaging the electron density near several sequential C^α^ positions by applying a transformation corresponding to the relationship between sequential residues in an α-helix (Kleywegt & Jones, 1997 ▶). The key element in this approach is that the C^β^ atoms in an α-helix point somewhat towards the N-terminus of the α-­helix and this directionality of the side-chain density can be readily identified after averaging over several sequential residues in a α-helix.

Here, we combine these methods for α-helix identification and placement and use them to create a simple series of steps for automatic modeling of the α-helices in an electron-density map of a protein.

## 2. Modelling α-helices in an electron-density map   

Our approach for modeling the α-helices in an electron-density map of a protein consists of three steps. These are as follows.

(i) Identification of α-helical density and modeling of α-­helical axes and extent using maps with varying low-resolution cutoffs.
(ii) Determination of α-helix placement (direction, rotation about and translation along the α-helical axis) using the full available resolution.
(iii) Assembly of α-helices, elimination of overlaps and joining of adjacent segments.

The result of this process is a model of the α-helical portions of the structure that can be used as a starting point for further model building and map interpretation. These steps are described in detail below.

### 2.1. Identification of α-helical density and modeling of α-­helical axes and extent using maps with varying low-resolution cutoffs   

**Figure 1:**
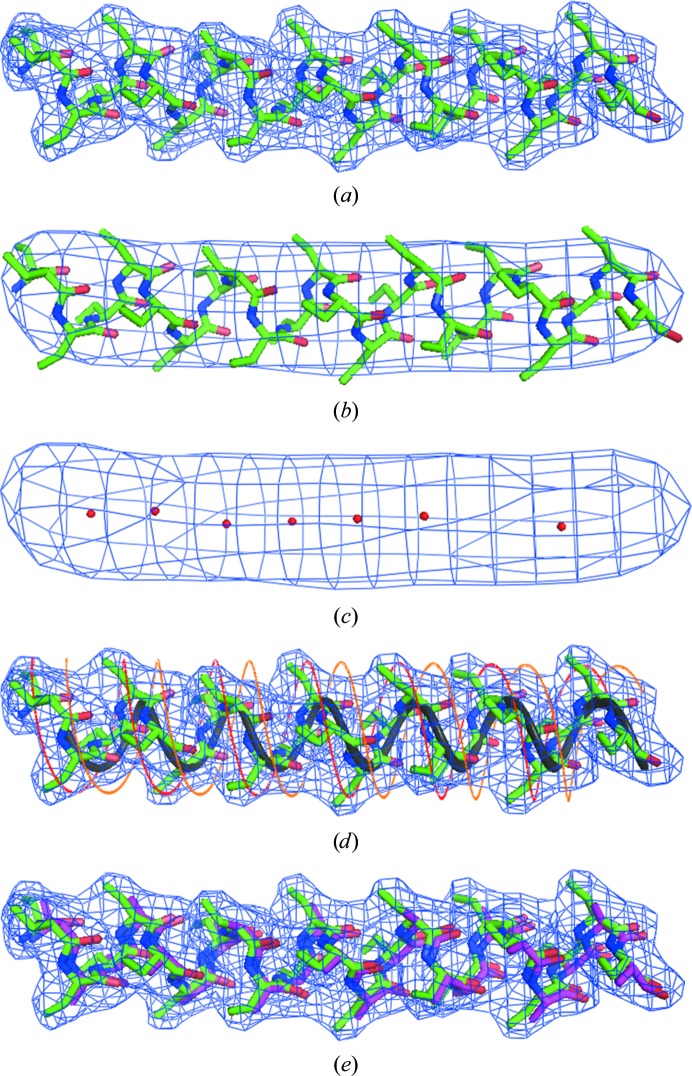
Model α-helix density and interpretation. (*a*) Model α-helix at a resolution of 3 Å. (*b*) Model α-helix at a resolution of 7 Å. (*c*) Points along the axis of a tube of density at a resolution of 7 Å. (*d*) Positioning an α-helix in model density. The dark blue mesh is a contour of model electron density at a resolution of 3 Å. The gray helix is fitted to the main-chain atoms of the model α-helix and has a radius of 2 Å and a pitch of 5.4 Å. The red and yellow helices are offset by ±1 Å along the helix axis from the gray main-chain helix and have radii of 4 Å. (*e*) Model α-helix (in green), model density (in blue) and fitted α-helix (in red). This figure was created using *PyMOL* (DeLano, 2002 ▶).

The first step in our process for modeling α-helices in the electron-density map of a protein is to identify the α-helices using a set of maps with low-resolution cutoffs from about 5 to 8 Å. While at high resolution an α-helix has a rather complicated pattern of density (Fig. 1 ▶ *a*), at a resolution of 7 Å an α-­helix appears as a tube of density (Fig. 1 ▶ *b*), so that finding the α-helices can be quite straightforward.

A map is calculated (typically with a grid of about 1/3 to 1/6 the resolution of the map) at low resolution (7 Å in Fig. 1 ▶ *b*) and a set of points is identified along the axis of the tubes of density corresponding to α-helices. The points are chosen to be a set for which (i) each point is in relatively high density (typically at least 2σ, where σ is the r.m.s. of the map), (ii) no more than one point that is adjacent to a chosen point has an electron-density value that is greater than the value at the chosen point and (iii) each chosen point is at least a specified distance (typically 2 Å) from each other chosen point. The second criterion is chosen to ensure that the chosen points are either at a peak of density or along a line of high density. A set of points satisfying these criteria for the map in Fig. 1 ▶(*b*) is shown in Fig. 1 ▶(*c*).

Next, the points along the axis of the tube of density as shown in Fig. 1 ▶(*c*) are used to guess the location and direction of the axis of the tube of density. Each point is considered as a possible marker of the center of a tube of density and the directions to every other point (typically including only those within 25 Å) are considered, one at a time, as the direction of the tube of density. The center and direction are scored by calculating the electron density at intervals of typically 2 Å along the line they define and identifying the longest segment that satisfies the criteria that (i) every point along the line has a density ρ of at least ρ_mean_ × cut_1_, where ρ_mean_ is the mean density in the segment and cut_1_ has a typical value of 0.5, and (ii) the points on the ends have densities of at least ρ_mean_ × cut_2_, where the value of cut_2_ is typically 0.75. These are the same criteria as used previously in building protein main-chain segments (Terwilliger, 2003 ▶). The score is then the square root of the number of points sampled along the line multplied by the mean: ρ_mean_ × *N* ^1/2^. For each point, the direction yielding the highest score is saved. An additional optimization of the direction is then carried out by sampling randomly chosen directions within approximately 30° of the saved direction. The overall highest scoring direction is then saved along with the extent of the segment in which the sampled points satisfied the two criteria. This yields a set of potential α-helix locations, orientations and ends.

The final step in low-resolution identification of α-helices is to score each potential α-helix based on the correlation of density between the low-resolution electron-density map and an idealized tube of positive density. The basic idea in this scoring is to ensure that the potential α-helices have high density down their axis and low density a few angstroms away from the axis, as would a tube of density. In this simple scoring scheme, the idealized density consists of a tube of density down the axis of the potential α-helix with a density of 1 on the axis and zero elsewhere. The correlation is calculated down the axis of the α-helix and on the surface of a cylinder with a radius of 4 Å and an axis coincident with the axis of the α-­helix. These correlations are then used to score each potential α-helix location, and the top-scring locations (typically those with a correlation coefficient cc_helix_min of 0.5 or greater) are saved.

This process is typically repeated with maps with resolution cutoffs from about 5–8 Å and all the resulting α-helices are considered in the following steps.

### 2.2. Determination of α-helix placement (direction, rotation about and translation along the helical axis) using the full available resolution   

The second overall step in α-helix identification is to use the high-resolution electron-density map to determine how an α-­helix could be optimally placed in the electron density given the helix axis and the ends of the helical segment. This is performed in three stages. Firstly, the positioning along the helix axis of the tubes of density in the map corresponding to the main-chain atoms in each (potential) helix is determined. The direction of the α-helix is then identified and finally the positioning of an idealized α-helix is identified.

Fig. 1 ▶(*d*) illustrates the approach used to position the helix axis of a segment in ideal α-helical density. The blue mesh corresponds to a contour of ideal density from an α-helical segment and the gray helix is an ideal helix with a radius of 2 Å and a pitch of 5.4 Å. The parameter that is optimized in this step is the translation of the gray ideal helix along the helix axis, with a score given by the mean density along the gray ideal helix multiplied by the square root of its length. As in the previous overall step, the ends of the helix are chosen to maximize its length, while requiring that the density at all intermediate points and at the ends be at least cut_1_ or cut_2_ times the mean in the segment, respectively.

The direction of the α-helix is identified by maximizing the density at the positions where C^β^ atoms would be located given the location of the gray helix representing main-chain atoms as identified above. Fig. 1 ▶(*d*) illustrates this process. Two helices (shown in red and yellow in Fig. 1 ▶ *d*) are constructed based on the gray helix. Each of these helices has a radius of 4 Å and a pitch of 5.4 Å. They are offset by ±1 Å along the helix axis from the gray main-chain helix. Depending on the direction of the helix, one of these two helices (the red helix in Fig. 1 ▶ *d*) will typically be in much higher average density than the other, allowing the direction of the helix to be identified. A *Z* score is estimated reflecting the confidence in this difference from the ratio of the difference between the scores for the two directions to the estimated standard deviation of this ratio for random helix placements. This standard deviation is estimated from the variance of the values of the scores obtained for both directions, assuming incorrect periodicities of a helix of 80°, 90°, 110° and 120°. If the *Z* score was 2 or larger, the assignment of the direction was considered to be likely to be correct.

The positioning and extent of an idealized polyalanine α-­helix in the high-resolution electron density is then identified by a simple search over rotations about the helix axis and translations along the helix axis, trimming the ends in the same fashion as described above and scoring by the mean value of electron density at the coordinates of atoms in the idealized α-­helix multiplied by the square root of the number of atoms. Fig. 1 ▶(*e*) shows the position of the model polymethylalanine α-­helix used to generate the density for Fig. 1 ▶ in green along with the positioning of the polyalanine α-helix carried out this way in orange.

### 2.3. Assembly of α-helices, elimination of overlaps and joining of adjacent segments   

The previous steps result in a collection of α-helices that match the electron density but that may contain overlapping or otherwise incompatible fragments of α-helix. The assembly of all these fragments and the resolution of overlaps is carried out by the main-chain assembly routines in the *RESOLVE* software (Terwilliger, 2003 ▶). This process consists of ranking all fragments (α-helices) based on their match to the density using the scoring function described above and identifying fragments that have two or more sequential C^α^ atoms that overlap within about 1 Å and that can therefore be connected into longer chains. The highest scoring chain is then selected and all overlapping fragments are deleted. This process is continued until no fragments of at least a minimum length (typically four residues) are found. The resulting set of α-­helices is saved.

## 3. Application to experimental electron-density maps   

**Figure 2:**
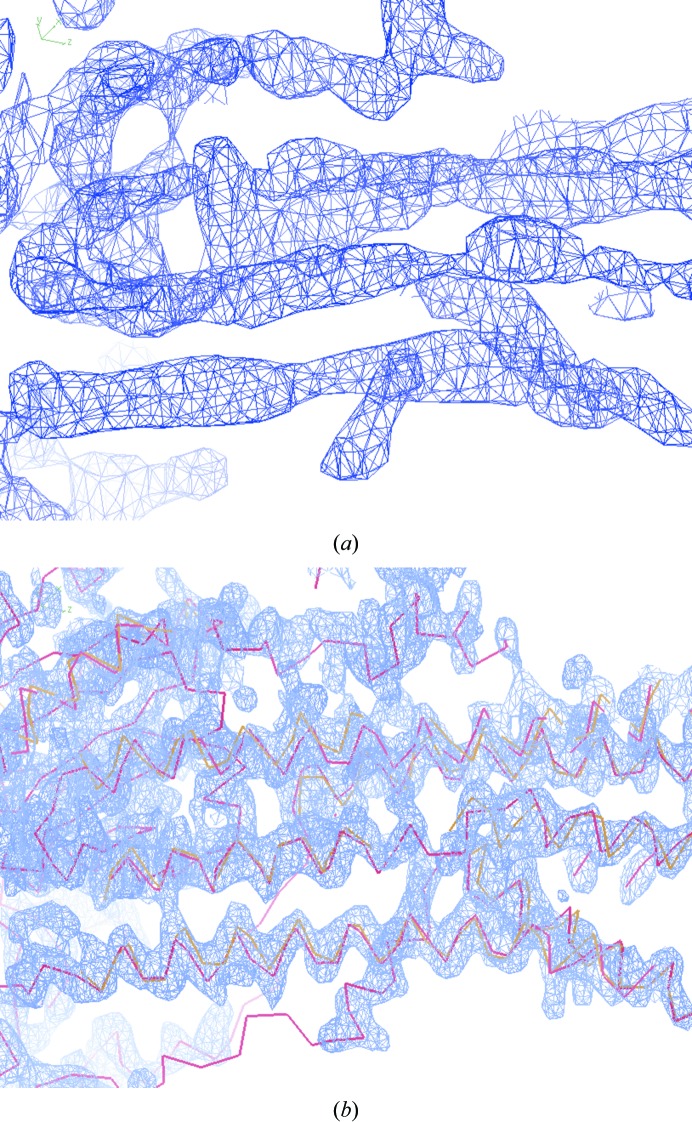
SAD-phased density-modified electron-density map of a calcium pump (Sorensen *et al.*, 2004 ▶) recalculated using the *PHENIX AutoSol* wizard at a resolution of 3.1 Å. (*a*) Section of map truncated at a resolution of 7 Å. (*b*) The same section as in (*a*) but calculated at a resolution of 3.1 Å, showing the helices found with the present procedure in yellow and those from the refined structure (PDB entry 1t5s; Sorensen *et al.*, 2004 ▶) in red. This figure was created using *Coot* (Emsley & Cowtan, 2004 ▶)

We first tested our algorithm for α-helix identification using the electron-density map of a calcium pump with a transmembrane segment consisting of α-helices (Sorensen *et al.*, 2004 ▶). For this analysis the map was recalculated using the *PHENIX AutoSol* wizard (Adams *et al.*, 2002 ▶; Terwilliger *et al.*, 2008 ▶) using SAD data to a resolution of 3.1 Å. A portion of this map truncated to a resolution of 7 Å is shown in Fig. 2 ▶(*a*). Tubes of density corresponding to helices are readily identifiable in the map. Fig. 2 ▶(*b*) shows the map at high resolution, along with the α-helices that were identified using the procedure described here (in yellow) and the α-helices from the refined structure (PDB entry 1t5s; Berman *et al.*, 2000 ▶; Bernstein *et al.*, 1977 ▶; Sorensen *et al.*, 2004 ▶) (in red). It can be seen that the C^α^ positions of the α-helices identified using the present method very closely match those in the refined structure.

We next applied the method to a set of 42 density-modified electron-density maps obtained with MAD, SAD, MIR and a combination of SAD and SIR procedures with data extending to high resolutions ranging from 1.5 to 3.8 Å. These maps were calculated with the *PHENIX AutoSol* wizard (Terwilliger *et al.*, 2008 ▶) using data that had previously led to refined models for each of the structures considered. Each map was examined for α-helices using the procedure described above.

Table 1 ▶ summarizes the results of these tests, listing for each structure the number of residues of α-helix in the refined structure (as calculated with *DSSP*; Kabsch & Sander, 1983 ▶), the number of residues of α-helix found, the number of these residues that were correctly placed in α-helices (with a C^α^ atom within 3 Å of a C^α^ atom in an α-helix in the refined structure), the quality of the map (the correlation of the map with a map calculated from the refined model of the structure), the r.m.s. coordinate difference between main-chain atoms in the modeled α-helices compared with those in the refined structure and the correlation between the map and a map calculated from the α-helix model.

Overall, 63% of the 11 233 residues in α-helices in the refined structures were found. Viewed differently, 76% of the residues that were built using the present method in fact corresponded to α-helical segments of the refined structures, with a C^α^ atom within 3 Å of a C^α^ atom in an α-helix in the refined structure. The remaining 24% were built into structure that was not identified as α-helical by *DSSP*. The overall r.m.s.d. between modeled α-helices and refined coordinates (matching the closest corresponding atom, *e.g.* C^α^ with C^α^, and including incorrectly modeled α-helices, but excluding any atoms more than 10 Å from any atom in the refined structures) was 1.3 Å. The CPU time (using 2.9 GHz Intel Xeon processors) required to analyze all 42 maps was 28 min or about 0.2 s per residue of α-helix placed. To provide a frame of reference for these results, we carried out one cycle of automated model building applying the *PHENIX AutoBuild* wizard (Terwilliger *et al.*, 2008 ▶) to the same maps as used above. This procedure includes *RESOLVE* model building and *phenix.refine* refinement. The *AutoBuild* wizard correctly built 75% of the 11 233 residues in α-helices in the refined structures with an overall r.m.s.d. (for all main-chain and C^β^ atoms in the entire models built) of 0.95 Å, requiring 43 h for the 42 maps.

**Figure 3:**
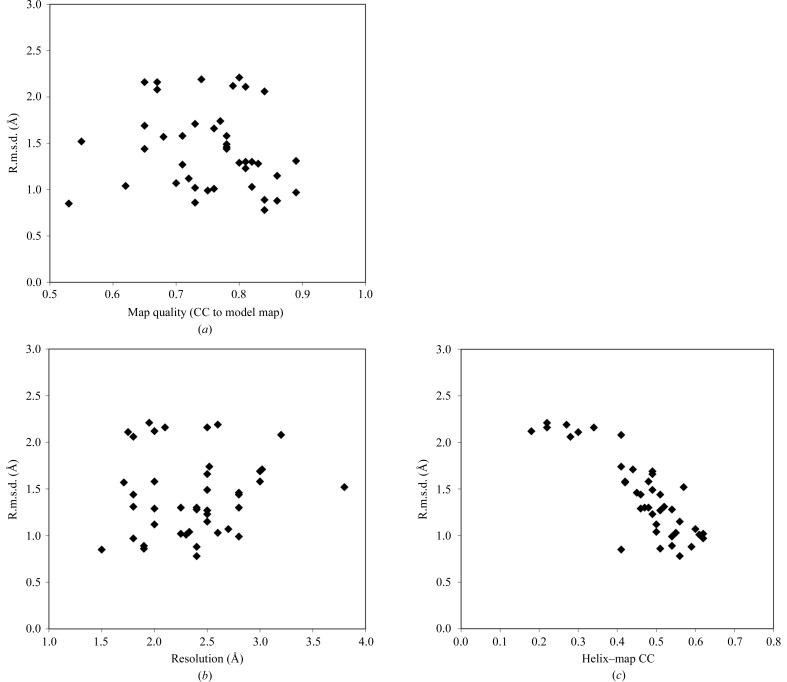
Accuracy of α-helical models. The r.m.s.d. between the α-helical models obtained using the present method and the corresponding refined models from Table 1 ▶ is plotted. (*a*) R.m.s.d. as a function of map quality. (*b*) R.m.s.d. as a function of resolution. (*c*) R.m.s.d. as a function of map–helical model correlation.

The maps used in this analysis were of fair to excellent quality, with correlations to model maps based on the corresponding refined structures of 0.53–0.89. Fig. 3 ▶(*a*) shows that for this set of maps the quality of the map has only a small effect on the quality of the α-helices built, as reflected in the r.m.s.d. between the main-chain atoms in the α-helices found and those in the corresponding refined models. Similarly, the resolution of the map, in the range 1.5–3.8 Å, had little effect on the quality of the models (Fig. 3 ▶ *b*). However, it was possible to tell which models were accurate. Fig. 3 ▶(*c*) shows that the map–model correlation based on the coordinates of the α-­helices that were built is inversely related to the r.m.s.d. between those coordinates and those of the corresponding refined structures. Those models with a model–map correlation of greater than about 0.45 generally had an r.m.s.d. of less than about 1.5 Å and those with lower model–map correlation generally had an r.m.s.d. of greater than 1.5 Å.

**Figure 4:**
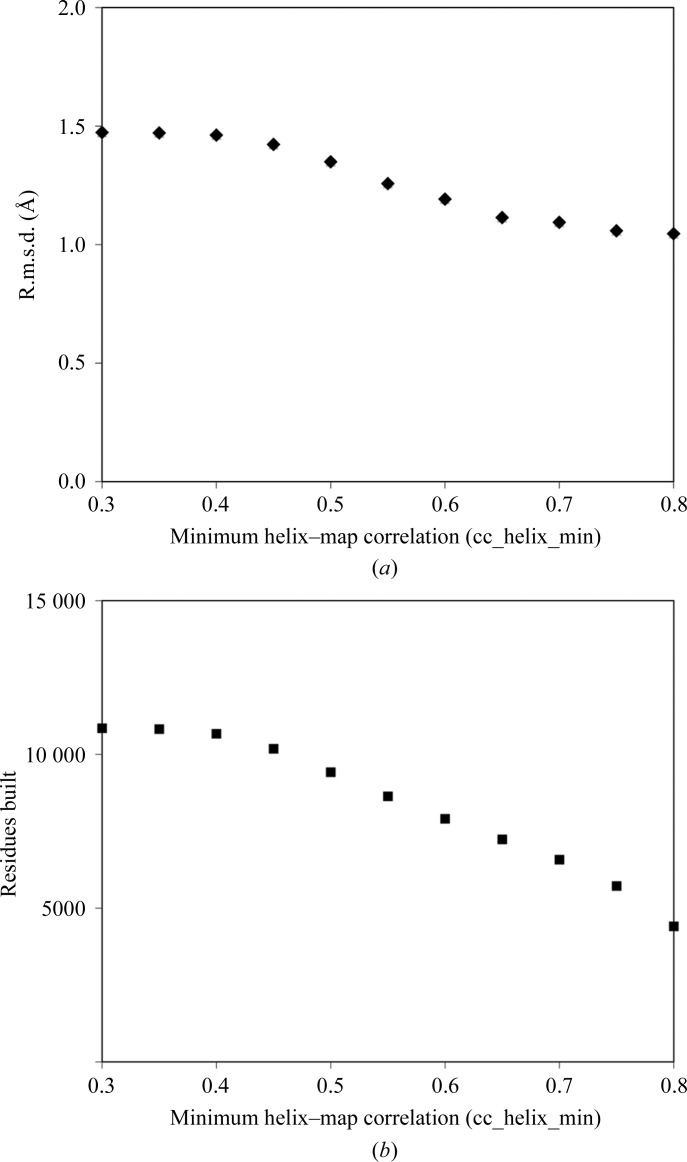
Accuracy and residues built *versus* cutoff for accepting helices. (*a*) The overall r.m.s.d. as in Fig. 3 ▶ is plotted as a function of the parameter cc_helix_min which defines the minimum correlation of density between a helix and the electron-density map. The default is 0.5. (*b*) The overall number of residues built for the 42 structures in Table 1 ▶ is plotted as a function of cc_helix_min.

One parameter that might be particularly important in determining both the accuracy of the procedure and the number of residues built is the map-correlation cutoff used to choose the density at low resolution (cc_helix_min). The default value is a correlation of 0.5. We tested a range of values of cc_helix_min for the set of 42 maps in Table 1 ▶. Fig. 4 ▶(*a*) shows the overall r.m.s.d. of main-chain atoms from those in corresponding refined models and Fig. 4 ▶(*b*) shows the total number of residues built. Increasing the threshold correlation results in more accurate models but fewer residues built and the default value of 0.5 appears to be a reasonable compromise between these effects.

## 4. Conclusions   

The procedure described here for the rapid placement of α-­helices in electron-density maps may be useful in several contexts. Firstly, it may be useful as a method for the evaluation of map quality. Secondly, it may be useful in giving a rapid indication to a crystallographer as to whether they have successfully determined the structure in their crystals. Thirdly, it may be a useful approach to generating a partial model of a protein that can then be extended with other model-building tools.

## Figures and Tables

**Table 1:** Helix identification in experimental electron-density maps

	Residues							
Structure	Total	Helix	Built	Correct	*d*_min_ ()	Map quality (CC to model map)	R.m.s.d. ()	Helixmap CC
RNase P (1nz0; Kazantsev *et al.*, 2003 ▶)	416	177	6	6	1.5	0.53	0.85	0.41
1063B (1lfp; Shin *et al.*, 2002 ▶)	243	92	65	58	1.7	0.68	1.57	0.42
Epsin (1edu; Hyman *et al.*, 2000 ▶)	149	100	98	83	1.8	0.89	0.97	0.62
Isocitrate lyase (1f61; Sharma *et al.*, 2000 ▶)	836	387	385	286	1.8	0.65	1.44	0.51
MBP (1ytt; Burling *et al.*, 1996 ▶)	227	42	30	17	1.8	0.89	1.31	0.52
P9 (1bkb; Peat *et al.*, 1998 ▶)	136	4	27	0	1.8	0.81	2.11	0.30
Penicillopepsin (3app; James Sielecki, 1983 ▶)	323	30	33	0	1.8	0.84	2.06	0.28
Myoglobin (Ana Gonzlez, personal communication)	154	110	59	54	1.9	0.73	0.86	0.51
ROP (1f4n; Willis *et al.*, 2000 ▶)	108	92	97	86	1.9	0.84	0.89	0.54
1167B (1s12; Shin *et al.*, 2005 ▶)	370	160	142	118	2.0	0.72	1.12	0.50
CobD (1kus; Cheong *et al.*, 2002 ▶)	355	129	61	45	2.0	0.80	1.29	0.46
NSF-N (1qcs; Yu *et al.*, 1999 ▶)	195	29	24	2	2.0	0.80	2.21	0.22
Synapsin (1auv; Esser *et al.*, 1998 ▶)	585	149	74	45	2.0	0.78	1.58	0.42
Tryparedoxin (1qk8; Alphey *et al.*, 1999 ▶)	143	40	8	0	2.0	0.79	2.12	0.18
PDZ (1kwa; Daniels *et al.*, 1998 ▶)	174	30	19	0	2.1	0.67	2.16	0.22
Fusion complex (1sfc; Sutton *et al.*, 1998 ▶)	867	789	716	702	2.3	0.73	1.02	0.62
GPATase (1ecf; Muchmore *et al.*, 1998 ▶)	992	318	191	129	2.3	0.82	1.30	0.48
Granulocyte (2gmf; Rozwarski *et al.*, 1996 ▶)	241	117	87	76	2.3	0.62	1.04	0.50
VMP (1l8w; Eicken *et al.*, 2002 ▶)	1141	654	621	528	2.3	0.76	1.01	0.61
Armadillo (3bct; Huber *et al.*, 1997 ▶)	457	329	232	197	2.4	0.86	0.88	0.59
Cyanase (1dw9; Walsh *et al.*, 2000 ▶)	1560	710	462	364	2.4	0.82	1.30	0.47
Mev kinase (1kkh; Yang *et al.*, 2002 ▶)	317	123	133	96	2.4	0.83	1.28	0.54
NSF D2 (1nsf; Yu *et al.*, 1998 ▶)	247	110	52	45	2.4	0.84	0.78	0.56
1102B (1l2f; Shin, Nguyen *et al.*, 2003 ▶)	344	118	137	79	2.5	0.78	1.49	0.49
AEP transaminase (1m32; Chen *et al.*, 2002 ▶)	2169	849	792	609	2.5	0.81	1.23	0.49
FLR (1bkj; Tanner *et al.*, 1996 ▶)	460	209	64	45	2.5	0.77	1.74	0.41
P32 (1p32; Jiang *et al.*, 1999 ▶)	529	190	235	172	2.5	0.86	1.15	0.56
PSD-95 (1jxm; Tavares *et al.*, 2001 ▶)	264	87	72	34	2.5	0.76	1.66	0.49
QAPRTase (1qpo; Sharma *et al.*, 1998 ▶)	1704	737	525	399	2.5	0.71	1.27	0.51
RNase S (1rge; Sevcik *et al.*, 1996 ▶)	192	23	32	11	2.5	0.65	2.16	0.34
Gene V (1vqb; Skinner *et al.*, 1994 ▶)	86	0	26	0	2.6	0.74	2.19	0.27
Rab3A (1zbd; Ostermeier Brnger, 1999 ▶)	301	110	104	89	2.6	0.82	1.03	0.55
GerE (1fse; Ducros *et al.*, 2001 ▶)	384	251	179	145	2.7	0.70	1.07	0.60
CP synthase (1l1e; Huang *et al.*, 2002 ▶)	534	220	186	150	2.8	0.75	0.99	0.54
Rh dehalogenase (1bn7; Newman *et al.*, 1999 ▶)	291	109	138	86	2.8	0.78	1.44	0.46
S-hydrolase (1a7a; Turner *et al.*, 1998 ▶)	861	349	343	240	2.8	0.81	1.30	0.48
UT synthase (1e8c; Gordon *et al.*, 2001 ▶)	990	306	293	180	2.8	0.78	1.46	0.45
1029B (1n0e; Chen *et al.*, 2004 ▶)	1130	379	255	116	3.0	0.73	1.71	0.44
1038B (1lql; Choi *et al.*, 2003 ▶)	1432	440	628	367	3.0	0.71	1.58	0.48
1071B (1nf2; Shin, Roberts *et al.*, 2003 ▶)	801	286	215	136	3.0	0.65	1.69	0.49
Synaptotagmin (1dqv; Sutton *et al.*, 1999 ▶)	275	8	71	3	3.2	0.67	2.08	0.41
GroEL (1oel; Braig *et al.*, 1995 ▶)	3668	1841	1443	1291	3.8	0.55	1.52	0.57
